# A prospective evaluation of a breast cancer prognosis signature in the observational RASTER study

**DOI:** 10.1002/ijc.28082

**Published:** 2013-03-04

**Authors:** CA Drukker, JM Bueno-de-Mesquita, VP Retèl, WH van Harten, H van Tinteren, J Wesseling, RMH Roumen, M Knauer, LJ van 't Veer, GS Sonke, EJT Rutgers, MJ van de Vijver, SC Linn

**Affiliations:** 1 Department of Surgical Oncology, Netherlands Cancer InstituteAmsterdam, The Netherlands; 2 Department of Pathology, Netherlands Cancer InstituteAmsterdam, The Netherlands; 3 Division of Psychosocial Research and Epidemiology, Netherlands Cancer InstituteAmsterdam, The Netherlands; 4 Department of Biometrics, Netherlands Cancer InstituteAmsterdam, The Netherlands; 5 Department of Surgery, Maxima Medical CentreEindhoven, The Netherlands; 6 Department of Surgery, Sisters of Charity Hospital and Cancer CenterLinz, Austria; 7 Agendia IncAmsterdam, The Netherlands; 8 Helen Diller Family Comprehensive Cancer Center, University of CaliforniaSan Francisco, CA; 9 Department of Medical Oncology, Netherlands Cancer InstituteAmsterdam, The Netherlands

**Keywords:** breast cancer, gene expression profiling, prognosis prediction, adjuvant systemic treatment

## Abstract

The 70-gene signature (MammaPrint™) has been developed on retrospective series of breast cancer patients to predict the risk of breast cancer distant metastases. The microarRAy-prognoSTics-in-breast-cancER (RASTER) study was the first study designed to prospectively evaluate the performance of the 70-gene signature, which result was available for 427 patients (cT1–3N0M0). Adjuvant systemic treatment decisions were based on the Dutch CBO 2004 guidelines, the 70-gene signature and doctors' and patients' preferences. Five-year distant-recurrence-free-interval (DRFI) probabilities were compared between subgroups based on the 70-gene signature and Adjuvant! Online (AOL) (10-year survival probability <90% was defined as high-risk). Median follow-up was 61.6 months. Fifteen percent (33/219) of the 70-gene signature low-risk patients received adjuvant chemotherapy (ACT) versus 81% (169/208) of the 70-gene signature high-risk patients. The 5-year DRFI probabilities for 70-gene signature low-risk (*n* = 219) and high-risk (*n* = 208) patients were 97.0% and 91.7%. The 5-year DRFI probabilities for AOL low-risk (*n* = 132) and high-risk (*n* = 295) patients were 96.7% and 93.4%. For 70-gene signature low-risk–AOL high-risk patients (*n* = 124), of whom 76% (*n* = 94) had not received ACT, 5-year DRFI was 98.4%. In the AOL high-risk group, 32% (94/295) less patients would be eligible to receive ACT if the 70-gene signature was used. In this prospective community-based observational study, the 5-year DRFI probabilities confirmed the additional prognostic value of the 70-gene signature to clinicopathological risk estimations such as AOL. Omission of adjuvant chemotherapy as judged appropriate by doctors and patients and instigated by a low-risk 70-gene signature result, appeared not to compromise outcome.

Over the last two decades breast cancer mortality has declined in Western countries. This decline has been ascribed to early detection due to the implementation of population-based mammographic screening programs and the introduction of adjuvant systemic therapy (AST).^1^ Fifty percent of all breast cancer patients are cured with loco-regional therapy alone, while the other 50% recur in the absence of AST. The combination of adjuvant chemotherapy and adjuvant endocrine therapy halves the breast cancer mortality rate throughout the first 15 years after diagnosis.^2^ Selection of those patients at high risk of relapse for AST is based on clinicopathological factors, such as age, tumor size, nodal status, histological grade and hormone receptor status. Current guidelines and clinical tools, such as Adjuvant! Online (AOL), use these factors to estimate the risk of recurrence and the benefit of AST. However, when using these standard clinicopathological factors, individual risk assessment remains challenging. Many women are treated with chemotherapy, without deriving significant benefit.^3^ To improve accuracy, several gene expression prognosis classifiers have been developed and validated on historic data to refine risk estimation based on current guidelines.^4^ One of these is the 70-gene signature (MammaPrint™), for which its accuracy to select the right patient for AST is being compared to the accuracy of AOL in a randomized trial called MINDACT, that completed accrual and primary results are awaited.^5^

Between 2004 and 2006, the 70-gene signature has been subjected to the first prospective study using a gene-expression prognosis classifier as a risk estimation tool, in addition to clinicopathological factors. The microarRAy prognoSTics in breast cancER (RASTER) study was conducted in 16 community hospitals in the Netherlands.^6^ The primary aim of this multicenter observational study was to assess the feasibility of implementing the 70-gene signature in a community-based setting and to study the clinical impact of the 70-gene signature test result on AST decision making.^6^ A secondary aim of the RASTER study was to assess the outcome of patients for whom a gene expression classifier was used to determine the need for adjuvant systemic treatment. Implementation of the 70-gene signature in daily clinical practice appeared feasible. A considerable discrepancy in risk estimations among different clinicopathological guidelines and the 70-gene signature was observed.^6^ The addition of the 70-gene signature test result to standard clinicopathological factors led to a change in AST advice in 19% of patients.^6^ Here, we report the 5-year follow-up data of the RASTER study.

## Patients and Methods

The RASTER study design, patient eligibility criteria and study logistics have been described elsewhere.^6^ In short, 812 female patients were enrolled after having given written informed consent. Four hundred twenty-seven patients were postoperatively eligible and for them a 70-gene signature (MammaPrint™, Agendia Inc., Amsterdam, The Netherlands) was obtained. All 427 patients were aged 18–61 years old and had a histologically confirmed unilateral, unifocal, primary operable, invasive adenocarcinoma of the breast (cT1–3N0M0). Exclusion criteria were a history of a malignancy (with exception of basal-cell carcinoma or cervical dysplasia) and neoadjuvant systemic treatment. After enrollment of 242 patients, the maximum allowed age was changed to 54 years, because the 70-gene signature had been developed in patients under 55 years of age. At that time, the validation of the prognostic value in patients aged over 55 years was not yet available.^7^

After enrollment, patients received surgery as their primary treatment. All patients underwent either breast conserving treatment or ablation of the breast. Within one hour after surgery, the tumor samples (stored without any preserving solution) were procured at the Pathology Department of the participating hospitals. To ensure routine clinical practice, the initial histopathology data were used for clinical risk assessment by the treating physician and in the statistical analysis, without central review of paraffin-embedded tumor samples. Details on tumor grading, assessment of hormone receptor status and HER2 status, RNA extraction and microarray analysis are described elsewhere.^8^,^9^ The RASTER study is registered on the International Standard Randomised Controlled Trial Register, number ISRCTN71917916. A summary of the study protocol is outlined online (http://www.controlled-trials.com/ISRCTN71917916).

### Established clinical risk classifications indexes

AST decisions in this study were based on the Dutch Institute of Healthcare Improvement (CBO) guidelines of 2004,^10^ the 70-gene signature result and doctors' and patients' preferences.

The CBO guidelines used between 2004 and 2006 were more restrictive in selecting patients for AST as compared to other guidelines and were primarily based on the assumption that adjuvant chemotherapy is only justified if an absolute survival benefit of more than 5% at ten years can be expected. According to the 2004 CBO guidelines, low clinical risk was defined as age over 35 years, tumor of grade 1 and 30 mm or smaller, grade 2 and 20 mm or smaller or grade 3 and 10 mm or smaller. Additionally, age less than 36 years with a grade 1 tumor of 10 mm or smaller was also defined as low risk. All other patients were defined as high risk. Notably, in the CBO guidelines, adjuvant endocrine treatment was advised only in clinically high-risk patients with hormone-receptor-positive tumors in combination with chemotherapy.^10^

To study how the addition of the 70-gene signature to a risk prediction tool used today influences clinical practice we used AOL software, version 8.0 to calculate 10-year survival probabilities based on the patient's age, tumor size, tumor grade, estrogen receptor status and nodal status.^11^,^12^ Patients were assigned to a high clinical risk if their calculated 10-year survival probability was less than 90%.^6^ In addition, sensitivity analyses were performed for different AOL cut offs ranging from 85% to 95%, including the cut off used for the MINDACT trial.^5^

### Statistical analysis

For this analysis, we estimated five-year distant-recurrence-free interval (DRFI), comprising distant recurrence and death from breast cancer. Overall survival (OS) and distant-disease-free-survival (DDFS) were also calculated.^13^ Survival curves were constructed using the Kaplan–Meier method and compared using the log-rank test. In case of ordinal variables (age, pT-stage of TNM, histological grade and nodal status) with more than two groups, we tested for trends (using the Cochran–Armitage test). A significant finding was defined as a *p*-value below 0.05. Analyses were performed using SAS version 9.2 and R version 2.14.0.

## Results

Follow-up data of all 427 patients who were enrolled in the RASTER study were updated until September 15, 2011. The first patient was enrolled January 22, 2004, the last patient December 18, 2006. Median follow-up was 61.6 months.

### Patient characteristics, AST and outcome stratified by 70-gene signature

Supporting Information Table 1 summarizes the patient characteristics defined by the result of the 70-gene signature as reported by Bueno-de-Mesquita *et al*.^6^ 70-gene signature high-risk patients more often had large, poorly differentiated, estrogen receptor (ER) negative, progesterone receptor (PR) negative and HER2 positive tumors than 70-gene signature low-risk patients. Nineteen percent (9/47) of invasive lobular breast cancer patients had a high-risk 70-gene signature, while 53% (183/345) of invasive ductal breast cancer patients had a high-risk 70-gene signature result. Twelve percent (16/136) of the grade 3 tumors were 70-gene signature low-risk, while 83% (72/87) of the grade 1 tumors were 70-gene signature low-risk. After a median follow-up time of 61.6 months, 24 DRFI events and 11 deaths occurred. Nine patients died due to breast cancer. One patient died due to lung cancer and one patient due to cardiac disease (Supporting Information Table 2). The 5-year DRFI probabilities for 70-gene signature low-risk (*n* = 219) and high-risk (*n* = 208) patients were 97.0% and 91.7% (*p* = 0.03), respectively (Supporting Information Fig. 1). Importantly, this difference in outcome was observed despite the fact that in the 70-gene signature low-risk group 15% (33/219) of the patients received adjuvant chemotherapy, versus 81% (169/208) in the high-risk group. The administered chemotherapy regimens for low- and high-risk patients are described in Supporting Information Table 1.

**Table 1:** Clinicopathological characteristics of patient groups defined by 70-gene signature (70-GS) and AOL risk estimations

		Total (*n* = 427)	70-GS low-AOL low (*n* = 95)	70-GS high-AOL low (*n* = 37)	70-GS low- AOL high (*n* = 124)	70-GS high-AOL high (*n* = 171)
Age	<35	26 (6%)	5 (5%)	0 (0%)	2 (2%)	19 (11%)
	36–40	41 (10%)	12 (13%)	7 (19%)	2 (2%)	20 (12%)
	41–45	84 (20%)	19 (20%)	14 (38%)	18 (14%)	33 (19%)
	46–50	141 (33%)	28 (30%)	8 (22%)	58 (47%)	47 (28%)
	51–55	100 (23%)	27 (28%)	8 (22%)	29 (23%)	36 (21%)
	>55	35 (8%)	4 (4%)	0 (0%)	15 (12%)	16 (9%)
pT (TNM)	pT1 (<20 mm)	301 (70%)	95 (100%)	37 (100%)	82 (66%)	87 (51%)
	pT2 (>20–50 mm)	125 (29%)	0 (0%)	0 (0%)	42 (33%)	83 (48%)
	pT3 (>50 mm)	1 (1%)	0 (0%)	0 (0%)	0 (0%)	1 (1%)
Histological grade	Good	87 (20%)	60 (63%)	12 (32%)	12 (10%)	3 (2%)
	Intermediate	204 (48%)	34 (36%)	19 (51%)	97 (78%)	54 (32%)
	Poor	136 (32%)	1 (1%)	6 (16%)	15 (12%)	114 (67%)
Histological type	Ductal	345 (81%)	73 (77%)	30 (81%)	89 (72%)	153 (89%)
	Lobular	47 (11%)	14 (15%)	2 (5%)	24 (19%)	7 (4%)
	Other	31 (7%)	7 (7%)	5 (13%)	9 (7%)	10 (6%)
	Unknown	4 (1%)	1 (1%)	0 (0%)	2 (2%)	1 (1%)
ER status	Negative	85 (20%)	0 (0%)	4 (11%)	3 (2%)	78 (46%)
	Positive	342 (80%)	95 (100%)	33 (89%)	121 (98%)	93 (54%)
PgR status	Negative	133 (31%)	9 (9%)	8 (21%)	24 (19%)	92 (54%)
	Positive	293 (69%)	86 (91%)	29 (78%)	100 (81%)	78 (46%)
	Unknown	1 (<1%)	0 (0%)	0 (0%)	0 (0%)	1 (1%)
HER2 status	Negative	358 (84%)	86 (91%)	29 (78%)	111 (90%)	132 (77%)
	Positive	48 (11%)	5 (5%)	5 (14%)	4 (3%)	34 (20%)
	Unknown	21 (5%)	4 (4%)	3 (8%)	9 (7%)	5 (3%)

**Table 2:** Kaplan–Meier risk estimations for DRFI and DDFS according to 70-gene signature and AOL stratification

70-Gene signature	AOL	AST	5-year DRFI (%) (95% CI)	5-years DDFS (%) (95% CI)
Low	Low	7/95 (7%)	95.3 (90.9–100)	94.3 (89.5–99.3)
High	Low	32/37 (86%)	100 (100–100)	94.6 (87.6–100)
Low	High	54/124 (44%)	98.4 (96.1–100)	97.6 (94.9–100)
High	High	166/171 (97%)	89.8 (85.1–94.8)	88.7 (83.8–93.8)

**Figure 1:**
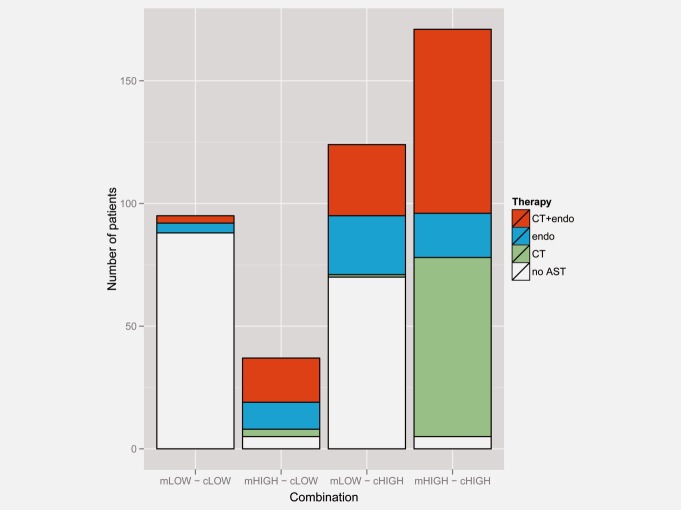
Distribution of patients (*n* = 427) over the four risk categories defined by 70-gene signature and AOL risk estimations and proportion and type of AST received per category. mLow = 70-gene signature low; mHigh = 70-gene signature high; cLow = AOL low; cHigh = AOL high; CT = adjuvant chemotherapy; Endo = adjuvant endocrine therapy; AST = adjuvant systemic therapy. [Color figure can be viewed in the online issue, which is available at http://wileyonlinelibrary.com.[

### Patient characteristics, AST and outcome stratified by 70-gene signature and AOL

Table 1 shows the patient characteristics stratified by 70-gene signature and AOL risk prediction. Discordant risk estimations between 70-gene signature and AOL occurred in 38% of the cases (161/427). Most discordant cases were 70-gene signature low-risk and AOL high-risk (124/427 = 29%), while 37 cases (37/427 = 9%) had a high-risk 70-gene signature result and a low-risk AOL estimation. Figure 1 summarizes the AST received in the different categories defined by 70-gene signature result and AOL. Ninety-three percent (88/95) of the patients who were 70-gene signature low-risk and AOL low-risk did not receive any AST (chemotherapy nor endocrine therapy). Fifty-six percent (70/124) of the patients who were 70-gene signature low-risk and AOL high-risk did not receive any AST. In Supporting Information Figure 1 Kaplan–Meier plots for DRFI, DDFS and OS are given for the whole group of patients, according to 70-gene signature, and according to AOL risk estimation. The 5-year DRFI probabilities for AOL low-risk (*n* = 132) and high-risk (*n* = 295) patients were 96.7% and 93.4%, respectively (*p* = 0.24) (Supporting Information Fig. 1). Table 2 shows DRFI and DDFS probabilities according to the combined risk categories.

The difference in OS outcome between 70-gene signature low-risk and AOL low-risk is partly due to the two cases, who died of non-breast cancer causes (Supporting Information Table 2) and who were categorized as 70-gene signature low-risk and AOL high-risk.

Sensitivity analyses were performed for different AOL cut offs ranging from 85 to 95%, showing a shift in the proportion of low risk patients without a substantial effect on DRFI, DDFS or OS survival probabilities (Supporting Information Table 3).

**Table 3:** Clinicopathological characteristics of 70-gene signature low risk patients who received no AST or ET only

		70-GS low- AOL low		70-GS low-AOL high	
			
		No AST (*n* = 88)	No AST or ET only (*n* = 92)	No AST (*n* = 70)	No AST or ET only (*n* = 94)
Age	<35	2 (2%)	3 (3%)	0 (0%)	0 (0%)
	36–40	11 (12%)	11 (12%)	0 (0%)	1 (1%)
	41–45	19 (22%)	19 (21%)	8 (11%)	8 (9%)
	46–50	26 (30%)	28 (30%)	32 (46%)	44 (47%)
	51–55	26 (30%)	27 (29%)	18 (26%)	26 (28%)
	>55	4 (5%)	4 (4%)	12 (17%)	15 (16%)
pT (TNM)	pT1 (<20 mm)	88 (100%)	92 (100%)	62 (89%)	75 (80%)
	pT2 (>20–50 mm)	0 (0%)	0 (0%)	8 (11%)	19 (20%)
	pT3 (>50 mm)	0 (0%)	0 (0%)	0 (0%)	0 (0%)
Histological grade	Good	57 (65%)	60 (65%)	8 (11%)	9 (10%)
	Intermediate	30 (34%)	31 (34%)	60 (86%)	77 (82%)
	Poor	1 (1%)	1 (1%)	2 (3%)	8 (9%)
Histological type	Ductal	68 (77%)	72 (78%)	47 (67%)	68 (72%)
	Lobulair	13 (14%)	13 (14%)	16 (23%)	19 (20%)
	Other	7 (8%)	7 (8%)	6 (9%)	6 (6%)
	Unknown	0 (0%)	0 (0%)	1 (1%)	1 (1%)
ER status	Negative	0 (0%)	0 (0%)	2 (3%)	2 (2%)
	Positive	88 (100%)	92 (100%)	68 (97%)	92 (98%)
PgR status	Negative	9 (10%)	9 (10%)	15 (21%)	21 (22%)
	Positive	79 (90%)	83 (90%)	55 (79%)	73 (78%)
	Unknown	0 (0%)	0 (0%)	0 (0%)	1 (1%)
HER2 status	Negative	79 (90%)	83 (90%)	63 (90%)	85 (90%)
	Positive	5 (6%)	5 (5%)	2 (3%)	2 (2%)
	Unknown	4 (4%)	4 (4%)	5 (7%)	7 (7%)

### Outcome at five years according to AST in patients with a low-risk 70-gene signature result

**Figure 2:**
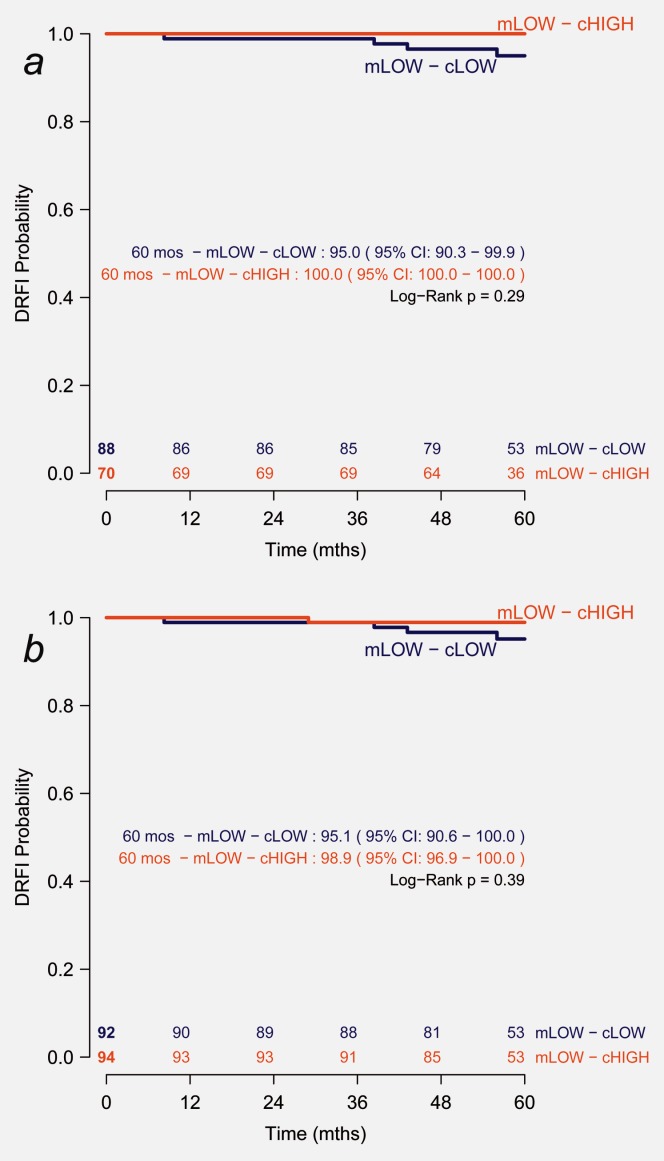
Five-year outcome of a) AST-naïve and b) chemotherapy-naïve patients with a low risk 70-gene signature result.

Five-year DRFI was 98.4% in patients with 70-gene signature low-risk—AOL high-risk (*n* = 124), of which 76% (*n* = 94) had not received adjuvant chemotherapy. The group that had not received adjuvant chemotherapy had a 5-year DRFI of 98.9%. The group that did not receive any systemic therapy (chemotherapy nor endocrine therapy) (*n* = 70) had a 5-year DRFI of 100% (Figs. 2*a* and 2*b*). No significant difference (*p* = 0.29) was seen between systemically untreated patients with a concordant low risk assessment and patients with a 70-gene signature low-risk result even with a high risk assessment by AOL. Table 3 shows the patient characteristics of patients who had a low risk 70-gene signature result and who had received adjuvant endocrine therapy only or no AST at all, split by AOL risk assessment.

## Discussion

The RASTER study provides the first prospective data on the outcome of patients with breast cancer for whom a gene expression prognosis classifier was used to determine the need for adjuvant systemic treatment. This community-based observational study confirms the potential of the 70-gene signature towards better selection of breast cancer patients who can forego adjuvant chemotherapy without compromising outcome. Use of the 70-gene signature reduced the proportion of high-risk patients as classified by AOL by 20% (87/427). In the AOL high-risk group, 32% (94/295) less patients would have received ACT if they had been treated according to the 70-gene signature risk estimation.

Overall, the 5-year outcome of the whole cohort was favorable, taking into consideration that 39% (168/427) had not received any form of AST. Most importantly, the 5-year DRFI probabilities were excellent for patients who were clinically high risk but had a low-risk 70-gene signature, even in the absence of AST. In addition, there was no difference in DRFI between 70-gene signature low-risk patients who were either clinical high or low risk.

Patients with a high-risk AOL result, but a low-risk 70-gene signature result who did not receive any AST (Table 2) more often had ER positive tumors with less often poor but more often intermediate histological grade than the total group of study patients. This group of patients had a 100% DRFI at five years.

One limitation of the comparison between the gene signature and AOL is that the actual treatment decisions in this study were based on the restrictive Dutch guidelines of 2004 and doctor's and patients' preferences. While this reflected clinical practice at the time of the study, equality of prognosis between groups that did or did not receive chemotherapy cannot be guaranteed. Subtle selection mechanisms may therefore have influenced our results. The reduction in the number of patients eligible for AST when using the 70-gene signature can also be explained by the definition of low risk that was used for AOL. The cut-off we used here (≥90% OS probability at ten years is defined as low risk), which is also used in the Dutch national guidelines of 2012, classifies a relatively large proportion of patients as high risk. A lower AOL cut-off (≥85%) results in more low risk patients and thus fewer patients who require AST. Despite this lower cut-off, the outcome of patients in the AOL low risk group remained excellent. To our knowledge a cut-off below 90% is thus far rarely used in clinical practice.

Another possible limitation is that AOL risk estimations are based on 10-year outcomes, whereas we report on 5-year outcomes. The prognostic capacity of the 70-gene signature is best at a follow-up time of five years and has less discriminatory power in years 5–10.^14^ From recent Oxford Overview data it is known that the carry-over effect of adjuvant chemotherapy gradually fades after five years.^2^,^15^,^16^ Therefore, the data in this study can be considered relatively mature for the effect of adjuvant chemotherapy on outcome. The carry-over effect of five year adjuvant endocrine therapy remains present at ten years of follow-up.^2^,^17^ Thus, the data presented here is immature regarding the effect of adjuvant endocrine therapy on long-term outcome and needs to be reevaluated at 10-years of follow-up. Consequently, only the effect on outcome of the decision to omit adjuvant chemotherapy based on a low-risk 70-gene signature can be derived from the current study.

Theoretically, the best survival for the entire group of breast cancer patients will be obtained by offering AST to all patients, as long as our prognostic tests are not 100% accurate.^18^ The mortality rate as a consequence of adjuvant chemotherapy toxicity is in the range of 1%.^19^ For adjuvant endocrine therapy, this is in the order of 0.3%. Hence, the real question is how many unnecessary deaths we are generally accepting by erroneously foregoing AST based on a false low-risk estimation to spare the large majority of breast cancer patients the unnecessary toxicity of adjuvant chemotherapy and consequent deterioration in quality of life based on a true low risk estimation.^20^ In this study, seven patients who developed distant metastases were low risk according to the 70-gene signature. Four of these patients were also low risk according to AOL. The other three patients were high risk according to AOL. One of these patients received both chemotherapy and endocrine therapy, one received endocrine therapy only, and one received no treatment at all. However, this AST untreated case developed a distant metastasis after 5 years (at 82 months of follow-up). Since 94 patients who had a 70-gene signature low risk/AOL high-risk result did not receive chemotherapy and had a 98.9% (95%CI: 96.9–100%; Fig. 2*b*) 5-year DRFI, one might infer that it costs about one avoidable distant recurrence (1.1%; 95%CI: 0–3.1%) to spare up to 94 patients unnecessary chemotherapy side-effects. When discussing the acceptable numbers-needed-to-treat and numbers-needed-to-harm, any prognostic factor that can improve the equation should be taken into account. The current data confirms that the 70-gene signature is such a prognostic factor.

In conclusion, in a prospective community-based observational study, the 5-year follow-up data confirmed the additional prognostic value of the 70-gene signature to clinicopathological factors used in AOL risk estimations. Omission of chemotherapy as judged appropriate by doctors and patients and supported by a low-risk 70-gene signature result appeared not to compromise outcome.

## What's new?

The “MammaPrint” is a 70-gene signature developed to predict the risk of breast cancer metastases. This study, the RASTER study, provides the first prospective data looking at this 70-gene signature to evaluate it's performance. For 427 patients, treatment decisions were based on standard guidelines, the 70-gene signature, and doctors' and patients' preferences. Here, 124 patients were categorized as “low-risk” by the 70-gene signature, but high-risk by other measures, such as age, tumor size, nodal status, and other clinicopathological factors. Of these, 76% did not receive adjuvant chemotherapy, and 98% survived 5 years with no recurrence of disease. Thus, withholding chemotherapy based on the low-risk gene signature result, and in accordance with doctors' and patients' preferences, did not negatively impact recurrence rate, confirming the prognostic value of this new tool.

## Authors Contribution

S.C.L., M.J.v.d.V., W.H.v.H. and L.J.v.V. were responsible for the study design and development of the protocol. W.H.v.H. ensured financing. The funding source had no role in the study design, data collection, data analysis, data interpretation, in writing the report, or in the decision to submit for publication. J.M.B.d.M. coordinated the study. E.J.T.R. and R.M.H.R. participated in the patient accrual. J.M.B.d.M., V.P.R., M.K. and C.A.D. took part in data collection. M.J.v.d.V., J.M.B.d.M. and J.W. took part in collection and processing of tumor samples. H.v.T. and G.S.S. performed the data analysis. C.A.D., G.S.S., E.J.T.R. and S.C.L. took part in data interpretation and manuscript writing. All authors were involved in reviewing the report.

## Principal and Co-Investigators of RASTER Study

The following clinicians entered patients and/or participated in the study (between the brackets, the number of accrued patients is mentioned): J. Meijer, J. Klinkenbijl, J. Douma, Alysis Care Group, Arnhem (31); J. Wijsman, D. van der Meer, P. de Wit, O. Loosveld, Amphia Hospital, Breda (4); S. Veltkamp, A. Baan, G. Timmers, K. van der Hoeven, Amstelland Hospital, Amstelveen (66); J. van der Bijl, A.M. Lenssen, I. Snijders, M. Nap, J. Wals, M. Pannebakker, Atrium Medical Center, Heerlen (13); L. Strobbe, F. van den Wildenberg, R. Berry, B. Dekker, E. Thunnissen, A. Uyterlinde, C. Mandigers, Canisius-Wilhelmina Hospital, Nijmegen (21); J.W. Arends, H. de Vries, A. Hemels-van der Lans, A. Imholz, Deventer Hospital, Deventer (40); I. Burgmans, C.I. Perre, T. van Dalen, J. van Gorp, D. ten Bokkel Huinink, P. Thunissen, Diakonessenhuis, Utrecht (4); J. Roussel, C. Bernhart, E. Weltevreden, S. Radema, Gelre Hospitals, Apeldoorn (21); R. Roumen, P. Reemst, A. Brands, K. Vercoelen, M. van Beek, W. Dercksen, G. Vreugdenhil, Maxima Medical Centre, Eindhoven/Veldhoven (114); T. van der Sluis, A. Stam, Lotus Sterk, Medisch Spectrum Twente, Enschede (6); M.J. Baas-Vrancken Peeters, H. Oldenburg, I. Eekhout, H. Hauer, J. Schornagel, Netherlands Cancer Institute-Antoni van Leeuwenhoek Hospital, Amsterdam (172); H. van der Mijle, D. de Vries, I. Kruithof, S. Hovenga, Nij Smellinghe Hospital; Drachten (18); B. de Valk, M. de Boer, P.J. Borgstein, A. Walter, Onze Lieve Vrouwe Gasthuis, Amsterdam (16); C. van Krimpen, P.W. de Graaf, C. van de Pol, N. van Holsteijn, A. van Leeuwen, M.M.E.M. Bos, E. Maartense, Reinier de Graaf Group, Delft (124); A. Zeillemaker, G. van Leeuwen, J. Calame, W. Molendijk, G. Jonkers, F. Cluitmans, Rijnland Hospital, Leiderdorp (59); and F. Bellot, G. Heuff, A. Tanka, P. Hoekstra, K. van de Stadt, J. Schrama, Spaarne Hospital, Hoofddorp (103).

## References

[1] Esserman LJ, Shieh Y, Rutgers EJ (2011). Impact of mammographic screening on the detection of good and poor prognosis breast cancers. Breast Cancer Res Treat.

[2] Early Breast Cancer Trialists' Collaborative Group (EBCTCG) (2005). Effects of chemotherapy and hormonal therapy for early breast cancer on recurrence and 15-year survival: an overview of the randomised trials. Lancet.

[3] Bedard PL, Cardoso F (2011). Can some patients avoid adjuvant chemotherapy for early-stage breast cancer?. Nat Rev Clin Oncol.

[4] Ross JS, Hatzis C, Symmans WF (2008). Commercialized multigene predictors of clinical outcome for breast cancer. Oncologist.

[5] Bogaerts J, Cardoso F, Buyse M (2006). Gene signature evaluation as a prognostic tool: challenges in the design of the MINDACT trial. Nat Clin Pract Oncol.

[6] Bueno-de-Mesquita JM, van Harten WH, Retel VP (2007). Use of 70-gene signature to predict prognosis of patients with node-negative breast cancer: a prospective community-based feasibility study (RASTER). Lancet Oncol.

[7] Mook S, Schmidt MK, Weigelt B (2010). The 70-gene prognosis signature predicts early metastasis in breast cancer patients between 55 and 70 years of age. Ann Oncol.

[8] Glas AM, Floore A, Delahaye LJ (2006). Converting a breast cancer microarray signature into a high-throughput diagnostic test. BMC Genomics.

[9] van de Vijver MJ, He YD, van't Veer LJ (2002). A gene-expression signature as a predictor of survival in breast cancer. N Engl J Med.

[10] Kwaliteitsinstituut voor de Gezondheidszorg CBO VvlK (2004).

[11] Olivotto IA, Bajdik CD, Ravdin PM (2005). Population-based validation of the prognostic model ADJUVANT! for early breast cancer. J Clin Oncol.

[12] Ravdin PM, Siminoff LA, Davis GJ (2001). Computer program to assist in making decisions about adjuvant therapy for women with early breast cancer. J Clin Oncol.

[13] Hudis CA, Barlow WE, Costantino JP (2007). Proposal for standardized definitions for efficacy end points in adjuvant breast cancer trials: the STEEP system. J Clin Oncol.

[14] Bueno-de-Mesquita JM, Linn SC, Keijzer R (2009). Validation of 70-gene prognosis signature in node-negative breast cancer. Breast Cancer Res Treat.

[15] Clarke M, Coates AS, Darby SC (2008). Adjuvant chemotherapy in oestrogen-receptor-poor breast cancer: patient-level meta-analysis of randomised trials. Lancet.

[16] Peto R, Davies C, Godwin J (2012). Comparisons between different polychemotherapy regimens for early breast cancer: meta-analyses of long-term outcome among 100,000 women in 123 randomised trials. Lancet.

[17] Davies C, Godwin J, Gray R (2011). Relevance of breast cancer hormone receptors and other factors to the efficacy of adjuvant tamoxifen: patient-level meta-analysis of randomised trials. Lancet.

[18] Retel VP, Joore MA, Knauer M (2010). Cost-effectiveness of the 70-gene signature versus St. Gallen guidelines and Adjuvant Online for early breast cancer. Eur J Cancer.

[19] Colozza M, de Azambuja E, Cardoso F (2006). Breast cancer: achievements in adjuvant systemic therapies in the pre-genomic era. Oncologist.

[20] Park BW, Lee S, Lee AR (2011). Quality of life differences between younger and older breast cancer patients. J Breast Cancer.

