# Supplementary material for: A prospective evaluation of a breast cancer prognosis signature in the observational RASTER study
Source: Int J Cancer. 2013 Mar 4;133(4):929–36. doi: 10.1002/ijc.28082 (PMC3734625; doi:10.1002/ijc.28082)
Supplement: Supplementary file 1 [file ijc0133-0929-sd1.pdf]

Supplementary figure 1. Kaplan-Meier plots for DDFS, DRFI and OS by 70-gene signature or AOL

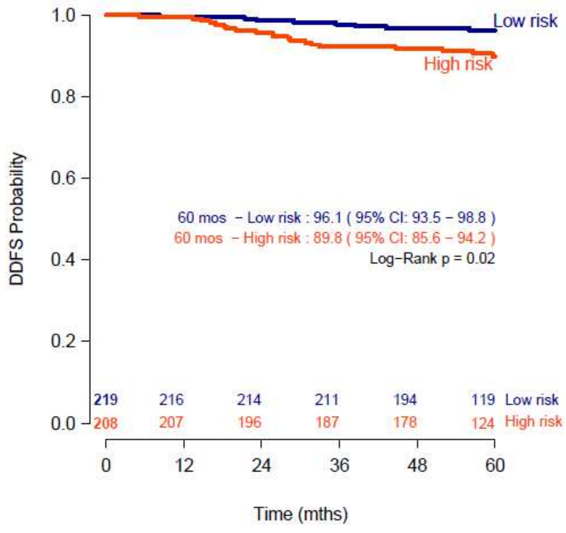

DDFS 70-gene signature

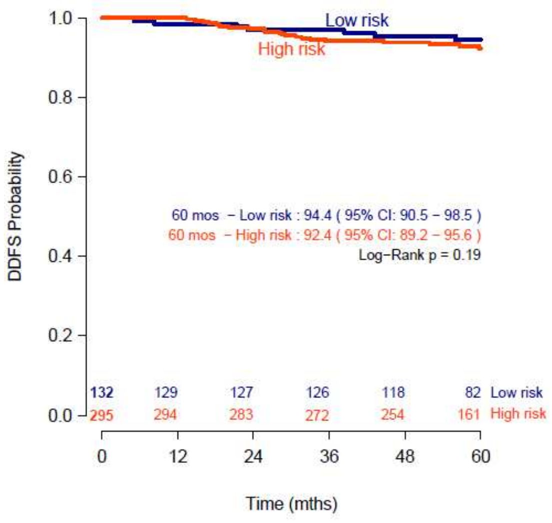

DDFS AOL

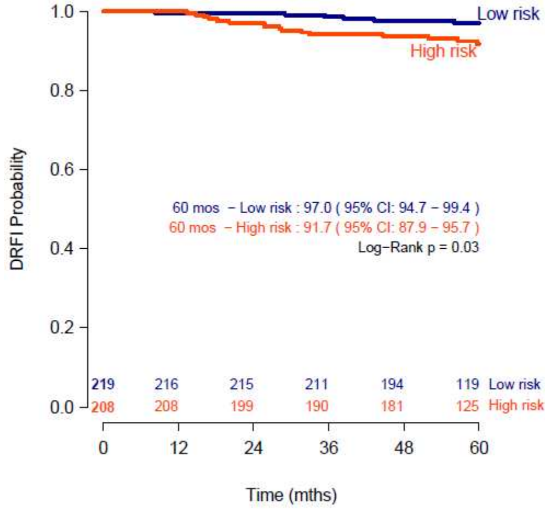

DRFI 70-gene signature

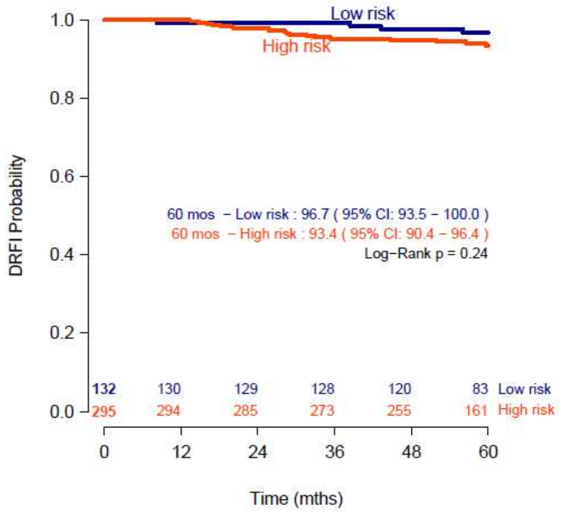

DRFI AOL

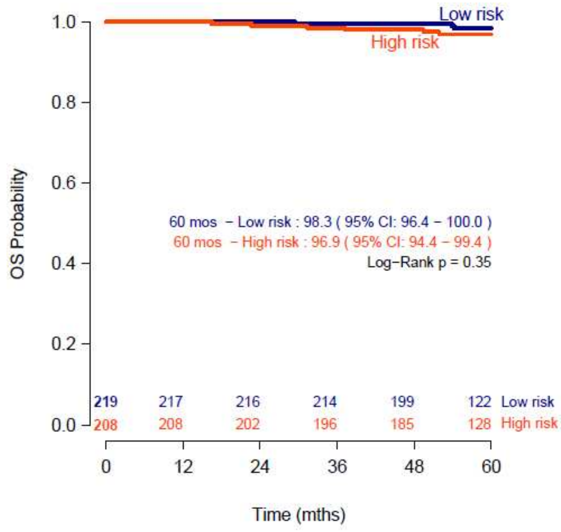

OS 70-gene signature

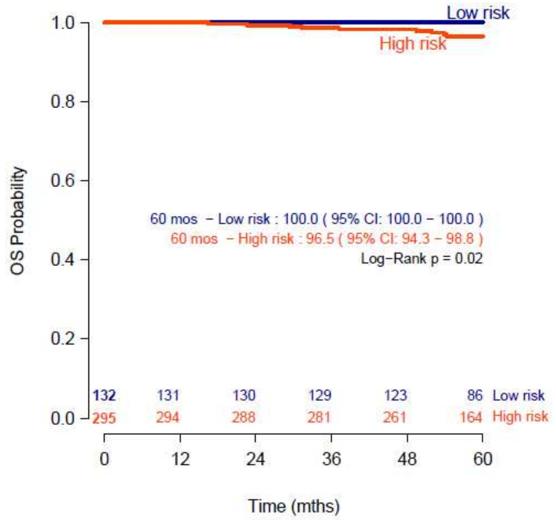

OS AOL

Supplementary table 1. Clinico-pathological characteristics by 70-gene signature result

		70-gene signature **Low Risk** (219)	70-gene signature **High Risk** (208)	**Total**
**Age**	<35	7 (3%)	19 (9%)	26 (6%)
	36-40	14 (6%)	27 (13%)	41 (10%)
	41-45	37 (17%)	47 (23%)	84 (20%)
	46-50	86 (39%)	55 (26%)	141 (33%)
	51-55	56 (26%)	44 (21%)	100 (23%)
	>55	19 (9%)	16 (8%)	35 (8%)
**pT (TNM)**	pT1 (<20mm)	177 (81%)	124 (60%)	301 (70%)
	pT2 (>20-50mm)	42 (19%)	83 (40%)	125 (29%)
	pT3 (>50mm)	0 (0%)	1 (0.5%)	1 (0.2%)
**Histological grade**	Good	72 (33%)	15 (7%)	87 (20%)
	Intermediate	131 (60%)	73 (35%)	204 (48%)
	Poor	16 (7%)	120 (58%)	136 (32%)
**Histological type**	Ductal	162 (74%)	183 (88%)	345 (81%)
	Lobular	38 (17%)	9 (4%)	47 (11%)
	Other	16 (7%)	15 (7%)	31 (7%)
	Unknown	3 (1%)	1 (0.5%)	4 (1%)
**ER status**	Negative	3 (1%)	82 (39%)	85 (20%)
	Positive	216 (99%)	126 (61%)	342 (80%)
**PgR status**	Negative	33 (15%)	100 (48%)	133 (31%)
	Positive	186 (85%)	107 (51.5%)	293 (68.6%)
	Unknown	0 (0%)	1 (0.5%)	1 (0.2%)
**HER2 status**	Negative	197 (90%)	161 (77%)	358 (84%)
	Positive	9 (4%)	39 (19%)	48 (11%)
	Unknown	13 (6%)	8 (4%)	21 (5%)
**CBO 2004**	Low Risk	167 (76%)	76 (37%)	243 (57%)
	High Risk	52 (24%)	132 (63%)	184 (43%)
**Adjuvant! Online**	Low Risk	95 (43%)	37 (18%)	132 (31%)
	High Risk	124 (57%)	171 (82%)	295 (69%)
**Chemotherapy**	None	186 (85%)	39 (19%)	225 (53%)
	FEC/FAC*	25 (11%)	108 (52%)	133 (31%)
	AC**	7 (3,5%)	26 (12%)	33 (8%)
	TAC***	0 (0%)	20 (10%)	20 (5%)
	AC-Paclitaxel	1 (0,5%)	15 (7%)	16 (4%)

*Chemotherapy regimen consisting of fluorouracil, cyclophosphamide and either adriamycine or epirubicine
** Adriamycine and cyclophosphamide
*** Docetaxel, adriamycine and cyclophosphamide

Supplementary table 2. Characteristics of patients with one or more events

Type of Event	IHC subtype	70-gene signature low risk	70-gene signature high risk
Locoregional event	ER+HER2-	4‡	3
	ER+HER2+	0	0
	ER-HER2-	0	1
	ER-HER2+	0	1
	ER+HER2 unknown	0	1
	Median age at diagnosis (range)	42 (27-46)	49 (33-59)
Distant metastasis event	ER+HER2-	6*	5
	ER+HER2+	1*	2#
	ER-HER2-	0	8
	ER-HER2+	0	2
	Median age at diagnosis (range)	46 (39-57)	50 (34-59)
Breast cancer-specific death	ER+HER2-	2	2
	ER+HER2+	0	0
	ER-HER2-	0	4
	ER-HER2+	0	1
	Median age at diagnosis (range)	ND (46-51)	51 (45-59)
Death due to other causes		2†	0
Contralateral breast cancer$^{\Delta}$		10‡	4
Second primary tumor		2	7

IHC = immunohistochemistry
‡One patient first developed an ipsilateral axillary recurrence, 10 months later followed by a contralateral breast cancer.
*Of these 7 70-gene signature®low risk cases, 4 cases were also low risk according to AOL. Of the three AOL high risk cases, only one did not receive any form of AST. This case developed a recurrence at 82 months of follow-up.
#One case was AOL low risk and 70-gene signature high risk. No AST was administered. She had a recurrence at 5 months of follow-up.
†One patient died due to a cardiac cause. This patient had only received adjuvant radiotherapy of the breast. She had no signs of breast cancer recurrence. She had had an ER+HER2- breast cancer at the age of 57. The second patient died of right-sided primary lung cancer, proven by histology and ER negative immunohistochemistry, two years after the primary diagnosis of invasive lobular breast cancer, ER+HER2-. She had only received adjuvant radiotherapy on the right breast after breast conserving therapy. She had stopped smoking one year before the diagnosis of breast cancer.
$^{\Delta}$Out of patients with low risk 70-gene signature, only one had received AST. Of 4 high risk 70-gene signature patients, 3 had received AST. One AML (no adjuvant chemotherapy) and one lung cancer in the 70-gene signature low risk group. Four lung cancers, 2 colorectal cancers and 1 carcinoid in the 70-gene signature high risk group.

Supplementary table 3. Kaplan-Meier risk estimations for DRFI using different AOL cut-offs

**AOL cut-off 10-years OS**	**AOL**	**Number of patients**	**5-years DRFI (%) (95% CI)**
85%	Low	253 (59.3%)	97 (95.1-99.5)
	High	174 (40.7%)	90 (85.8-94.9)
90%	Low	132 (30.9%)	97 (93.5-100)
	High	295 (69.1%)	93 (90.4-96.4)
88% (ER+) / 92% (ER-)	Low	194 (45.4%)	98 (88.0-95.0)
	High	233 (54.6%)	92 (96.0-100)
95%	Low	19 (4.4%)	100 (100-100)
	High	408 (95.6%)	94 (91.8-96.6)
